# Supplementary material for: Anomalously rotary polarization discovered in homochiral organic ferroelectrics
Source: Nat Commun. 2016 Nov 23;7:13635. doi: 10.1038/ncomms13635 (PMC5494196; doi:10.1038/ncomms13635)
Supplement: Supplementary Information — Supplementary Figures 1-14 and Supplementary Table 1 [file ncomms13635-s1.pdf]

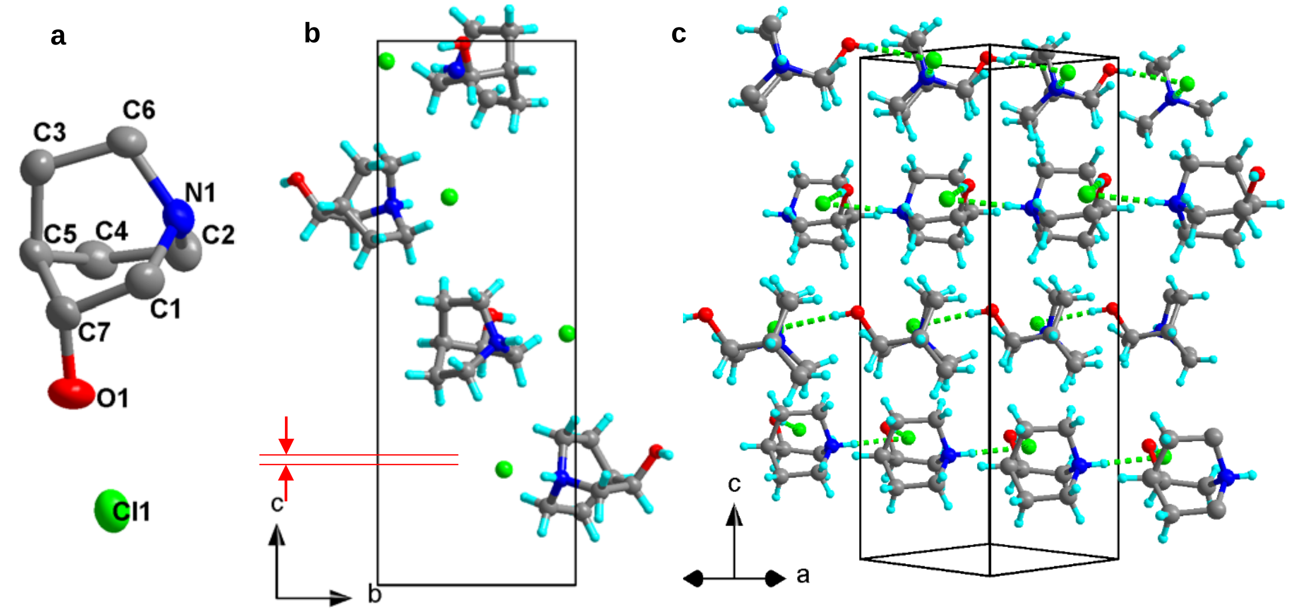

Supplementary Figure 1. Crystal structures of (*R*)-(-)-3-hydroxlyquinuclidinium (1). (**a**) A view of **1** with atomic numbering scheme. Displace ellipsoids were drawn at 50% probability level. H atoms were omitted for clarity. (**b**) The structural projection of **1** along the *a*-axis at room temperature, showing the structural helix and ferroelectric displacement. (**c**) The perspective view of **1**, showing the hydrogen bonding interactions.

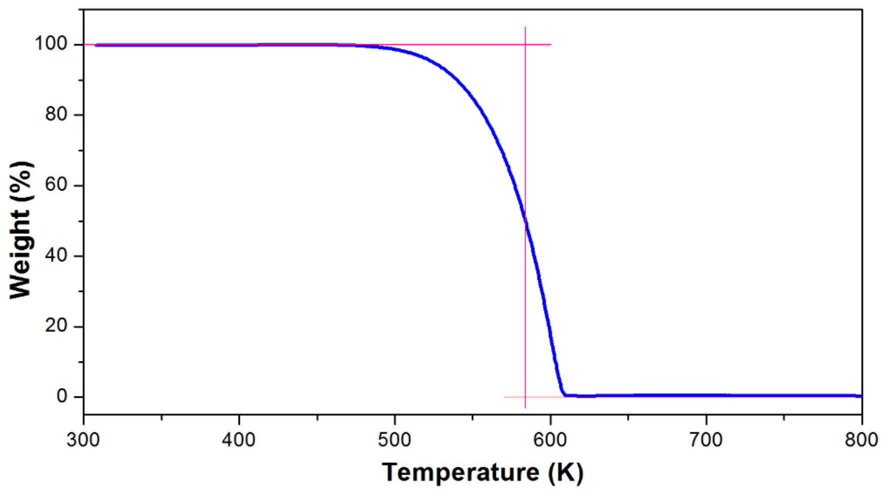

Supplementary Figure 2. The TGA result for (*R*)-(-)-3-hydroxlyquinuclidinium (1).

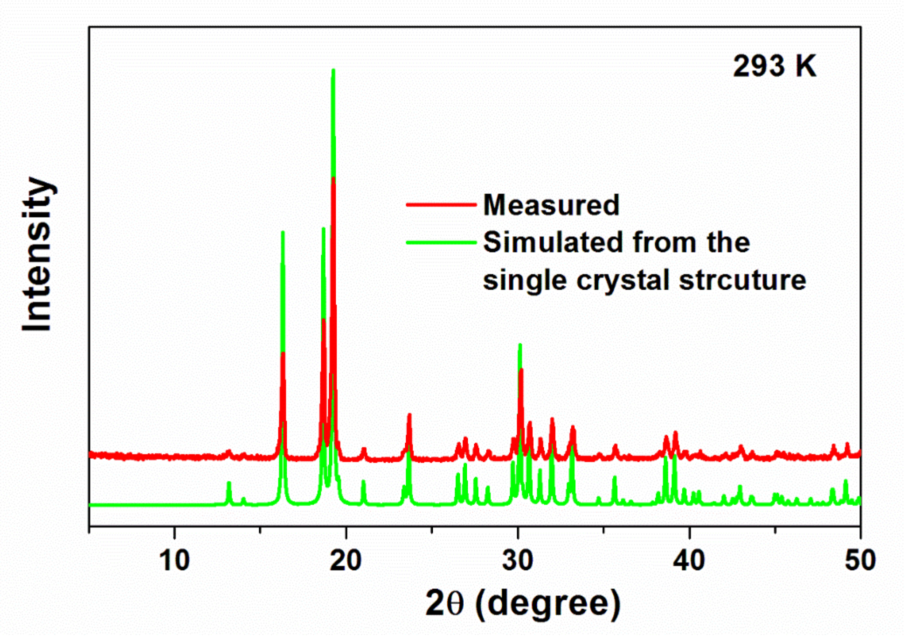

Supplementary Figure 3. The pattern of PXRD) of (*R*)-(-)-3-hydroxlyquinuclidinium (1).

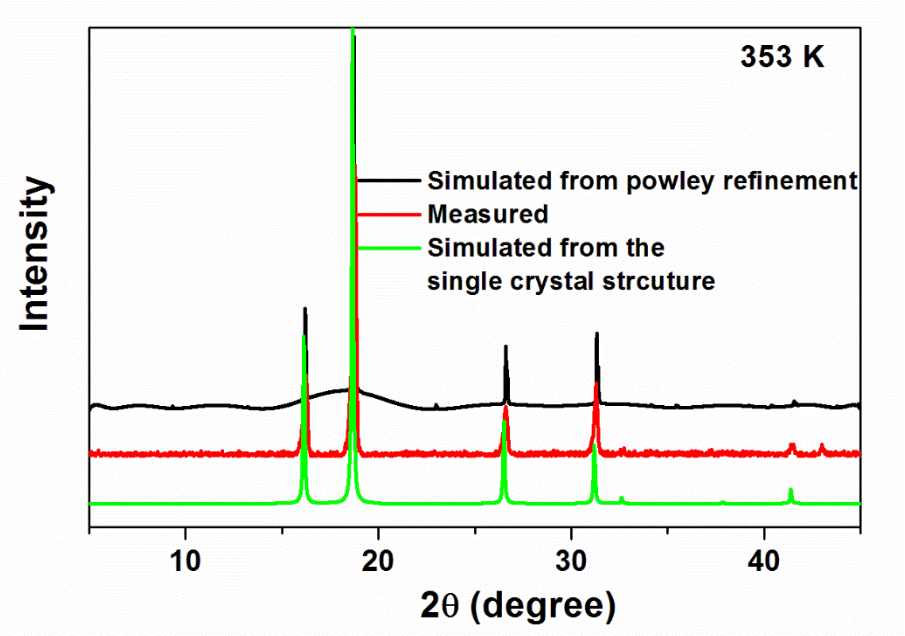

Supplementary Figure 4. Pawley refinement of PXRD data of 1. The refinement reveals a cubic unit cell of *a* = 9.4666 Å of the paraelectric phase. The pattern of PXRD data matches well from that simulated from the single crystal structure, supporting the structure model.

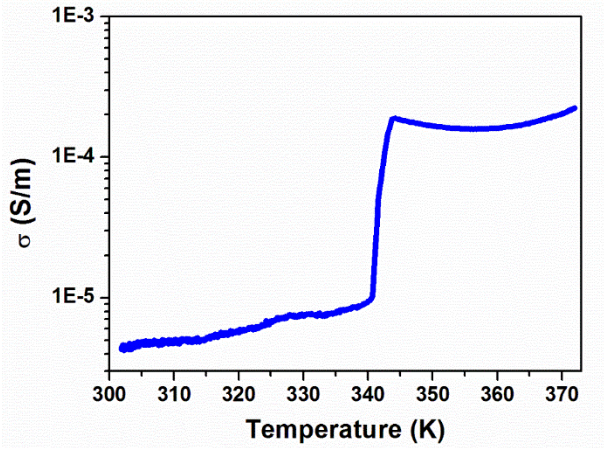

Supplementary Figure 5. The temperature-dependence of the conductivity of 1.

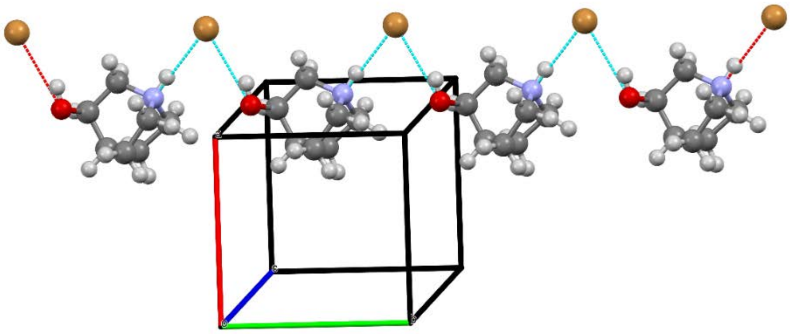

Supplementary Figure 6. The packing diagram of (*R*)-(-)-3-hydroxlyquinuclidinium bromide (2).

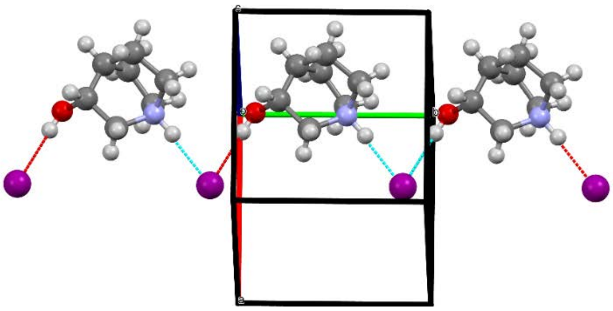

Supplementary Figure 7. The packing diagram of (*R*)-(-)-3-hydroxlyquinuclidinium iodide (3).

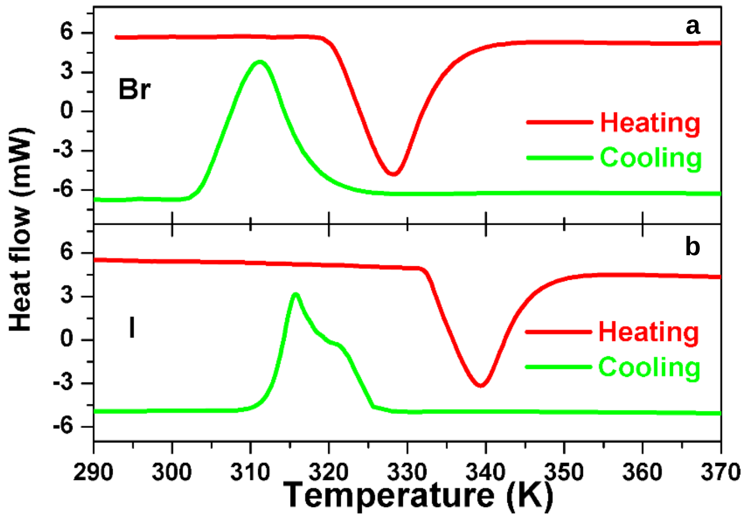

Supplementary Figure 8. DSC results for **2** and **3**. (**a**) The DSC curve for **2**, demonstrating the similar reversible phase transition to that in **1.** (**b**) The DSC curve for **3**, demonstrating the similar reversible phase transition to those in **1** and **2**.

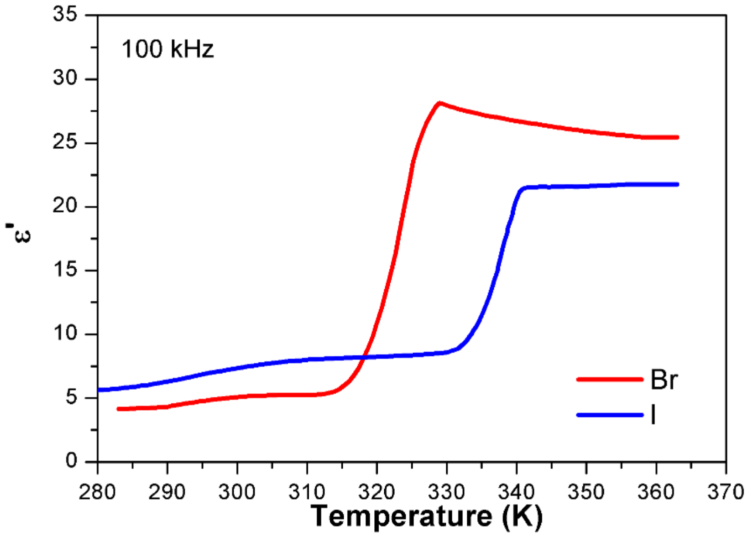

Supplementary Figure 9. Dielectric properties of 2 and 3. The results are for the polycrystalline samples.

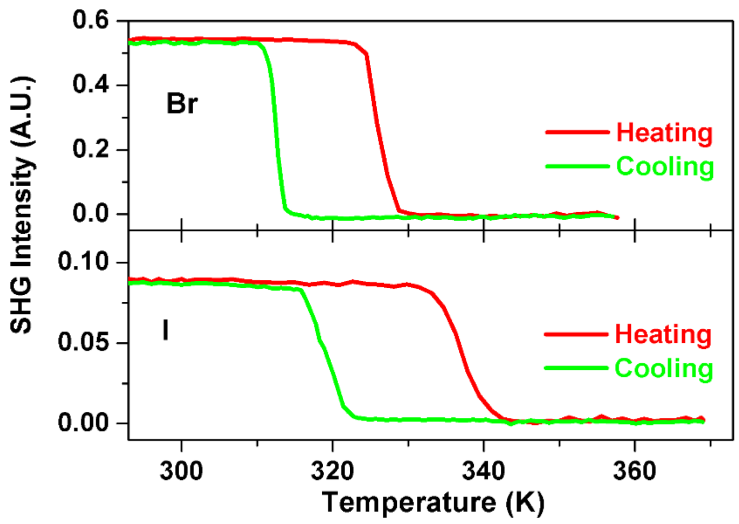

Supplementary Figure 10. SHG responses of 2 and 3. (**a**) The temperature-dependence of the SHG signal of **2**. (**b**) The temperature-dependence of the SHG signal of **3**.

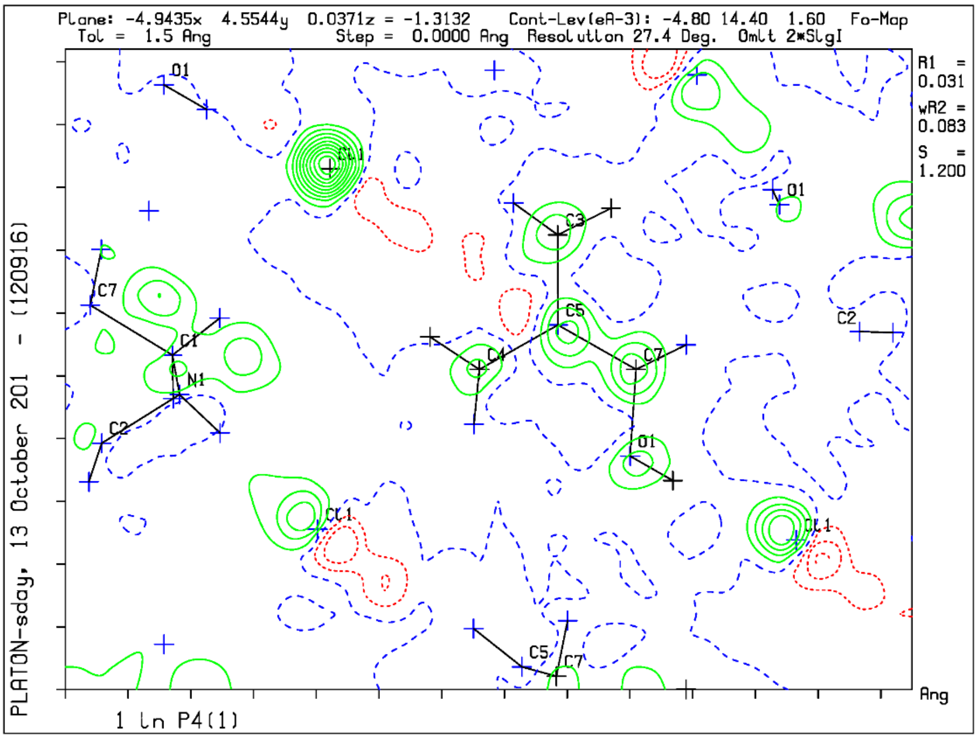

Supplementary Figure 11. The stereo image of a portion of the electron density map for 1 at 293 K.

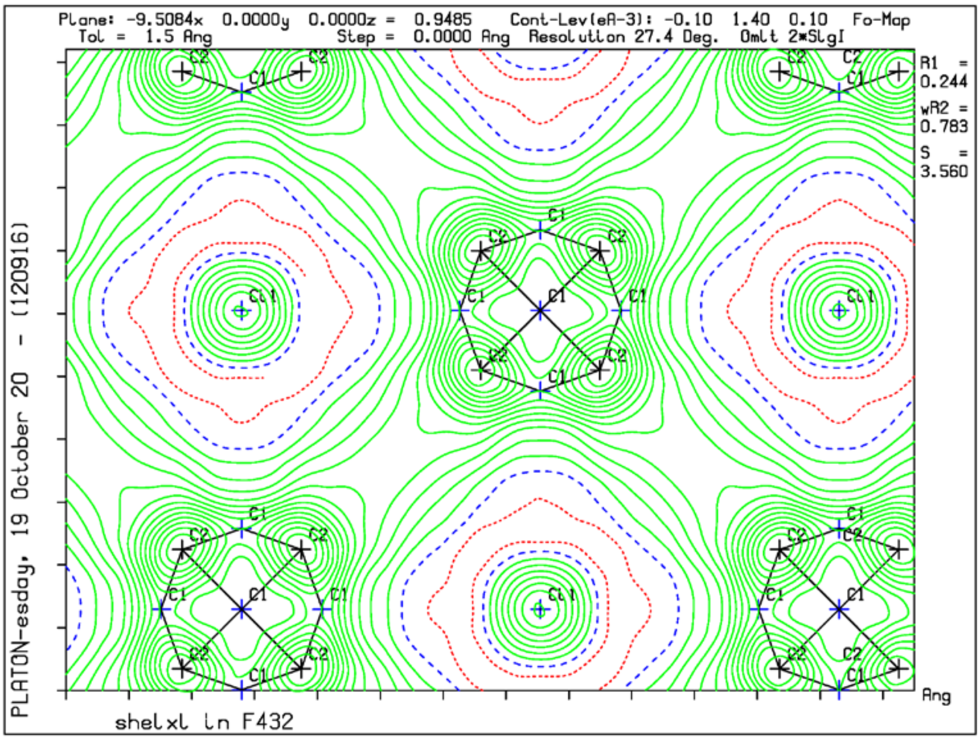

Supplementary Figure 12. The stereo image of a portion of the electron density map for 1 at 353 K.

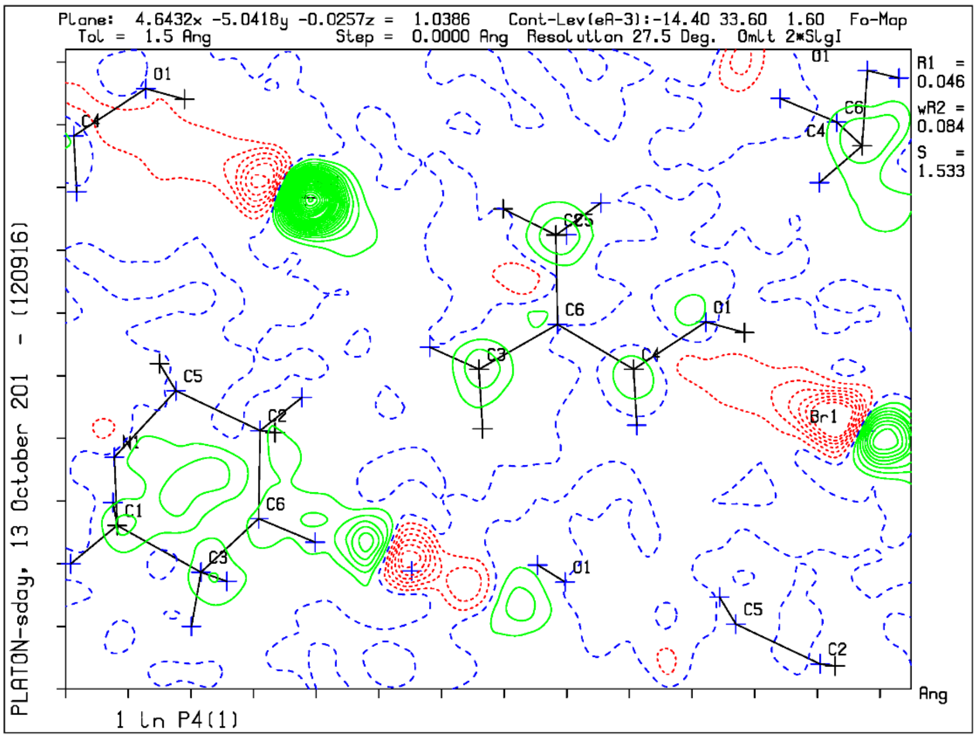

Supplementary Figure 13. The stereo image of a portion of the electron density map for 2 at 293 K.

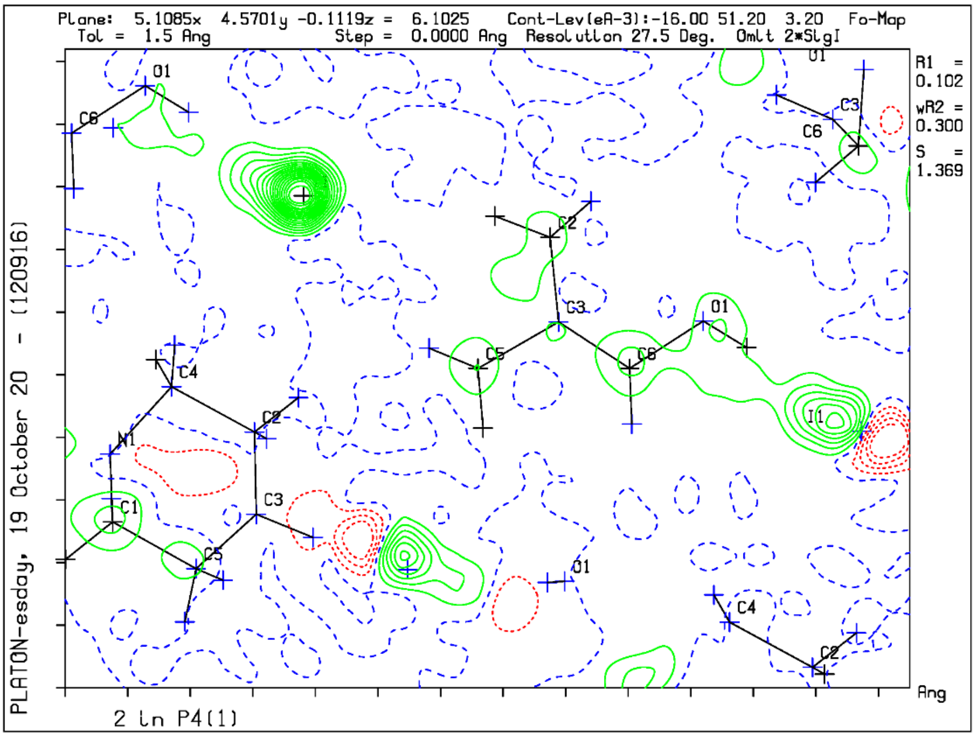

Supplementary Figure 14. The stereo image of a portion of the electron density map for 3 at 293 K.

Supplementary Table 1. Crystal data for (*R*)-(-)-3-hydroxlyquinuclidinium halides (1–3).

Compound	**1**		**2**	**3**
Chemical formula<br>formula wt	$C_7H_{14}ClNO$<br>163.64		$C_7H_{14}BrNO$<br>208.10	$C_7H_{14}INO$<br>255.09
Temperature (K)	293	353	293	293
Crystal system, space group	Tetragonal, *P*4$_1$	Cubic *F*432	Tetragonal, *P*4$_1$	Tetragonal, *P*4$_1$
a, *b*, *c* (Å)	6.7217(10)<br>6.7217(10)<br>18.464(4)	9.5084(18)<br>9.5084(18)<br>9.5084(18)	6.8541(10)<br>6.8541(10)<br>18.776(4)	6.8545(10)<br>6.8545(10)<br>18.778(4)
V (Å^3)	834.2(3)	859.7(5)	882.1(3)	882.3(3)
Dc (g cm^{-3})	1.303	1.264	1.567	1.920
μ (mm^{-1})	0.393	0.381	4.599	3.569
R_1 (I > 2σ(I))	0.0335	0.2488	0.0473	0.0948
wR_2 (all data)	0.0827	0.7024	0.0851	0.2763
S	1.099	3.45	1.134	1.155
